# MiR-29a Inhibits Glioma Tumorigenesis through a Negative Feedback Loop of TRAF4/Akt Signaling

**DOI:** 10.1155/2018/2461363

**Published:** 2018-08-14

**Authors:** Yongjie Liu, Naiquan Duan, Shibo Duan

**Affiliations:** ^1^Department of Neurosurgery, Cangzhou Central Hospital, Cangzhou, Hebei Province 061000, China; ^2^Department of Neurosurgery, Dacheng County Hospital, Dacheng, Hebei Province 065900, China

## Abstract

**Background:**

MiR-29a is known as a repressor of human cancer. However, its relevance in glioma proliferation and invasion remains largely unknown. In this study, we aimed to investigate the function and mechanism of miR-29a in glioma tumorigenesis.

**Methods:**

The expression of miR-29a was determined by using qRT-PCR. CCK-8, wound healing, and transwell invasion assays were carried out to analyze the effects of miR-29a in glioblastoma cells. qRT-PCR, luciferase reporter, and western blot experiments were done to validate the targeting of TRAF4/Akt pathway by miR-29a. The expression correlation between levels of TRAF4 and miR-29a was analyzed. Regulation of miR-29a expression by enhanced/reduced TRAF4/Akt expression was finally confirmed by qRT-PCR.

**Results:**

MiR-29a was decreased in the glioma tissues, especially in those at higher grades. Following its mimic transfection, we validated that miR-29a inhibited cell proliferation, migration, and invasion. Consistently, miR-29a inhibition induced the opposite effects on cell proliferation, migration, and invasion. We confirmed TRAF4 as a direct target of miR-29a, which might mediate the Akt pathway activation. We showed a significantly negative expression correlation between TRAF4 and miR-29a in normal and glioma tissues. Finally we observed an upregulation of miR-29a in TRAF4/Akt activated cells.

**Conclusion:**

MiR-29a is critical tumor suppressor for glioma tumorigenesis by forming a negative feedback loop of TRAF4/Akt signaling and represents a potent therapeutic candidate for treating gliomas.

## 1. Introduction

Among all the subtypes of cancers in brain and central nervous system (CNS), gliomas are the most frequent primary malignancies and the leading cause of deaths world widely [1]. Malignant gliomas, especially glioblastoma (GBM), are most aggressive and featured by fast growth and invasion into nearby brain tissue, thus leading to radical resection difficult for them [2, 3]. As a consequence, complete tumor resection is almost impossible, resulting in inevitable recurrence after surgery [4]. Now, growing molecular knowledge provides a broad range of modulations against this disease; however, the prognosis of GBM patients remains dismal, with a median survival time of 14.6 months [5, 6]. Therefore, it is urgent for us to identify new approaches of diagnosis, therapy, and prognosis for gliomas.

MicroRNAs (miRNAs) are short, highly conserved, and endogenous noncoding RNAs that repress target gene expression by inhibiting the translation or causing degradation of target mRNAs [7]. Regulation occurs through its binding to the 3'-untranslated region (3'-UTR) of the target gene mRNA [7]. A large quantity of evidence has suggested that miRNAs are critical regulators of nearly all physiological and pathological processes [8–10], including carcinogenesis of many types of human cancers such as gliomas [11]. Also, several miRNAs have been proved to be valuable prognostic biomarkers for glioma patients [12–14].

MiR-29 family has three closely related members, including miR-29a, miR-29b, and miR-29c [15]. Recent reports have found that miR-29s are important tumor suppressors and downregulated in multiple cancers including gliomas [16–18]. According to the known findings, the decreased miR-29s expression is closely associated with more aggressive phenotypes and shorter survival of glioma patients [18, 19], strongly indicating that they may serve as therapeutic candidates and prognostic biomarkers for gliomas. As a multitarget regulator, miR-29 contributes to various aspects of cancer cell behaviors such as proliferation, apoptosis, and invasion [17]. Although several targets have been reported for miR-29 [18, 19], we still should integrate more targets into the true function of miR-29 in cancer cells.

In this study, we confirmed that miR-29a downregulation caused TRAF4 and subsequent Akt activation in gliomas, and miR-29a itself inhibited GBM cell proliferation, migration, and invasion through directly targeting TRAF4. We also observed a negative feedback loop between miR-29a and TRAF4/Akt signaling, suggesting miR-29a as a potent therapeutic candidate for gliomas.

## 2. Materials and Methods

### 2.1. Patients and Tissues

The surgical specimens of 10 noncancerous brain tissues and 40 glioma samples were collected in Cangzhou Central Hospital from January 2014 to December 2017 with written consent. For total microRNAs extraction, tissues were frozen in liquid nitrogen immediately followed by storing in −80°C.

### 2.2. Cell Culture

Normal human astrocytes and human glioblastoma cell lines U118MG, U251, A172, and SNB19 were obtained from laboratory preservation. All cell lines were cultured in DMEM medium (Gibco, Life Technology) supplemented with 10% fetal bovine serum (Gibco, Life Technology) and 1% penicillin-streptomycin (Gibco, Life Technology). All the cells were cultured in a humidified atmosphere at 37°C under 5% CO2 condition.

### 2.3. MiRNA Mimics/Inhibitor and Cell Transfections

Oligo nucleotides miR-29a-3p mimics or inhibitors and scramble controls were purchased from GenePharma (Suzhou, China). Cells were transfected using Lipofectamine®RNAiMAX reagent purchased from Invitrogen as per the manufacturer's guidelines.

### 2.4. RNA Extraction and Quantitative Real-Time PCR (qRT-PCR)

Total RNA was extracted with Trizol Reagent (Invitrogen, Life Technology) as per the manufacturer's guidelines. The concentration and purity of all RNA samples were detected by a spectrophotometer. Special primers were used for the microRNA reverse transcription reaction, and U6B was used as an endogenous control of microRNAs. The oligo(dT)n primer was used for the reverse transcription reaction of cDNAs, and *β*-actin was used as an endogenous control of genes. The qRT-PCR assay was performed using the SYBR®Premix Ex Taq™ (Perfect Real Time) from Takara. Each sample was detected in triplicate and analyzed by the 2^−ΔΔCT^ method.

### 2.5. Cell Proliferation Assay

A172 or U251 cells were transfected with miR-29a mimics or inhibitors and control oligoes for 24h. Then the cells were seeded into 96-well plates with a density of 2×10^3^ cells/well and maintained overnight. On days 1-4, cell proliferation was measured by CCK-8 method (Dojindo, Kumamoto, Japan) by absorbance at 450nm. Three independent experiments were performed in quadruplicate.

### 2.6. Cell Migration Assay

Cell migration ability was determined using wound healing assay. The transfected A172 cells were seeded on a 6-well plate and grown until confluence. Then, a single scratch wound was generated with 1000*μ*l pipette tip. After gently washing with PBS for twice, the wounded cell monolayer was allowed to heal in a serum-free medium. The scratch wounds was photographed by inverted microscope and digital camera, followed by quantitated by using the ImageJ software. The result was shown as the percentage of wound closure setting the initial scratch width as 100%.

### 2.7. Invasion Assay

Cell invasion assay was performed using the 24-well Invasion Transwells (Corning) as per the manufacturer's guidelines. Transwell chambers were coated with BD Matrigel matrix as per the manufacturer's guidelines. 3×10^4^ U251 cells were seeded on top of the BD Matrigel in the upper chamber with serum-free medium, and the lower chamber was filled with the full culture medium containing 10% FBS. Cells that invade through the BD Matrigel-coated membrane after 36 hours were fixed with paraformaldehyde, followed by staining with 0.5% crystal violet solution and photographing under a microscope by counting six high-powered fields in the center of each well.

### 2.8. Western Blot

Proteins were extracted by using RIPA buffer (Beyotime, China) supplemented with 1mM PMSF (Beyotime, China). The protein concentration was measured by the BCA protein assay kit (Beyotime, China). A total of 40*μ*g of protein was isolated using 10% sodium dodecyl sulfate-polyacrylamide gel electrophoresis (SDS-PAGE) and transferred to a nitrocellulose (NC) membrane, followed by blocking nonspecific bands with 5% skimmed milk. Subsequently, the membrane was incubated with anti-TRAF4, anti-p-Akt, anti-Akt, or *β*-actin (Santa Cruz Biotechnology, CA, USA) primary antibodies overnight at 4°C, followed by washing 3 times with PBST buffer and incubation with a horseradish peroxidase (HRP) conjugated secondary antibody (Santa Cruz Biotechnology, CA, USA) for 1h at room temperature. Finally, the protein band was detected by an enhanced chemiluminescence (ECL) detection system (Pierce, Thermo Fisher Scientific).

### 2.9. Target Prediction and Dual-Luciferase Reporter Assays

Candidate targets of miR-29a-3p were predicted by free online tools of TargetScan (http://www.Targetscan.org/). The TRAF4 3′-UTR containing the predicted wild-type binding site for miR-29a-3p was amplified and cloned into pmirGLO vector (Promega Corporation). The TRAF4 3′-UTR containing mutant binding site for miR-29a-3p was subcloned by site-directed mutagenesis PCR method. 48h after transfection with miR-29a mimics, luciferase activity was detected using a dual-luciferase reporter assay system (Promega Corporation) and normalized to renilla activity.

### 2.10. Plasmids Construction

The pcDNA3.1-myc-his plasmid was from Life Technology Company. The coding sequence region of TRAF4 was amplified by PCR and subcloned into pcDNA3.1 plasmids with specific restriction enzymes.

### 2.11. Statistical Analysis

Statistical analysis was performed by using GraphPad 7.0. The differences were analyzed by Grouped Student's t-test. Data are presented as mean ± standard deviation (SD). A P value of <0.05 was considered to be statistically significant and a P value of <0.01 of extremely significant.

## 3. Results

### 3.1. MiR-29a Downexpression Correlates with Grades in Human Gliomas

**Figure 1:**
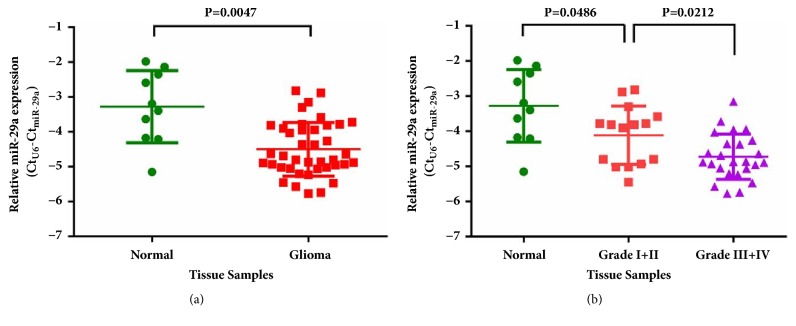
**Expression of miR-29a in noncancerous brain tissues and glioma samples. (a)** MiR-29a was decreased in glioma samples, compared with noncancerous brain tissues.** (b)** MiR-29a was decreased in glioma samples with high tumor grade (Grade I+II versus Grade III+IV).

To confirm the expression of miR-29a in human gliomas, we collected a total of 40 human gliomas and 10 nontumoral brain tissues. Using qRT-PCR, we detected expression levels of miR-29a and found that miR-29a was indeed downexpressed in gliomas (P<0.01; Figure 1(a)). Based on the WHO grades of these gliomas, we analyzed the miR-29a levels in Grades I-II (n=15) gliomas compared with Grades III-IV (n=25) gliomas and found that miR-29a was significantly decreased along with glioma grade elevation (P<0.05; Figure 1(b)). These results on the one hand confirmed the previous findings by others [18, 19] and on the other hand indicated the importance of miR-29a as a glioma repressor.

### 3.2. MiR-29a Inhibits GBM Cell Proliferation, Migration, and Invasion

**Figure 2:**
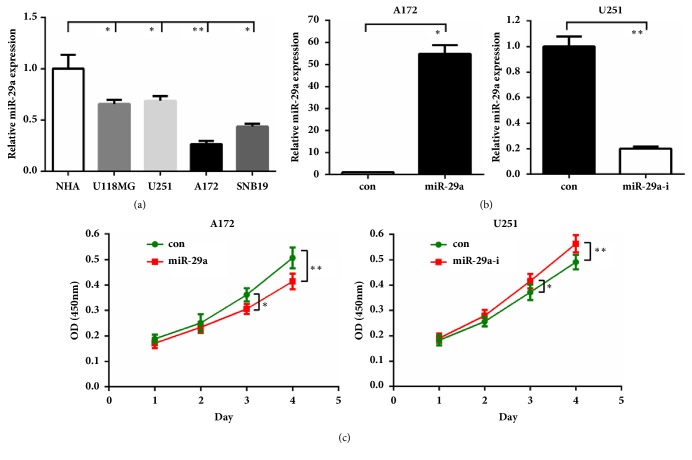
**MiR-29a inhibits glioma cell proliferation. (a) **MiR-29a was decreased in glioma cell lines, compared to normal human astrocytes.** (b)** A172 or U251 cells were transfected with miR-29a mimics or inhibitors. 48h later, cells were harvested for qRT-PCR.** (c)** CCK-8 assay was performed in A172 or U251 cells by using miR-29a mimics or inhibitors. *∗*:* P*<0.05; *∗∗*:* P*<0.01 versus control group.

To gain more insights of miR-29a's role in glioma, we then quantified its level in several GBM cell lines. Compared with the normal human astrocytes (NHA), miR-29a was downexpressed in the other four GBM cell lines (P<0.05~0.01; Figure 2(a)). Notably, miR-29a was the lowest expressed in A172 cells and somewhat higher in U251 cells. We then performed gain-of-function studies in A172 cells by overexpressing miR-29a mimics and loss-of-function studies in U251 cells by overexpressing miR-29a inhibitor. The transfection efficiency was verified by qRT-PCR (Figure 2(b)), then CCK-8 assay was carried out to evaluate the effects of miR-29 on GBM cell proliferation. As shown in Figure 2(c), overexpression of miR-29a led to reduced proliferation of A172 cells, whereas miR-29a inhibition caused elevated proliferation activity of U251 cells (P<0.05~0.01).

**Figure 3:**
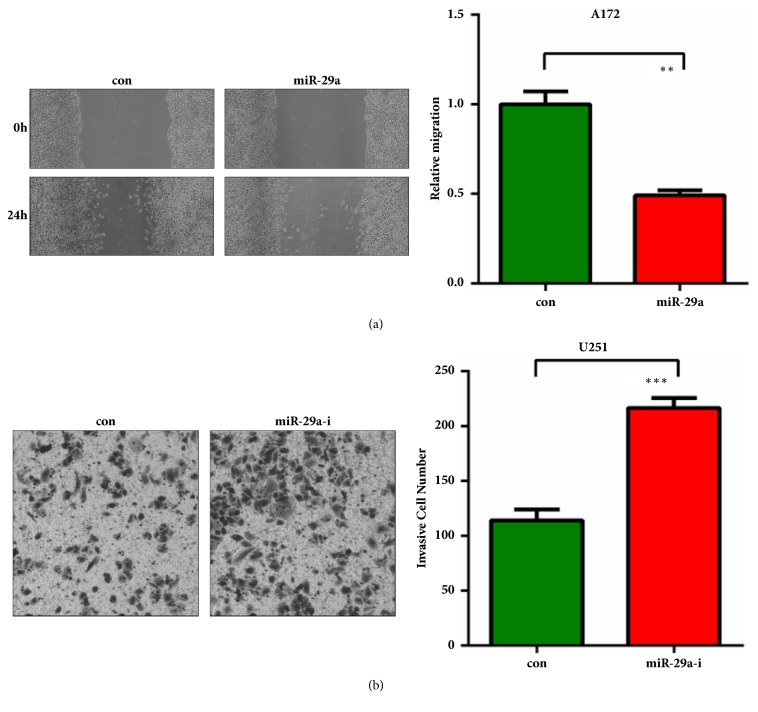
**MiR-29a inhibits glioma cell migration and invasion. (a) **A172 cells were transfected with miR-29a mimics with serum-free medium. 48h later, cells were harvested for wound healing assay.** (b) **U251 cells were transfected with miR-29a inhibitors with serum-free medium. 48h later, cells were harvested for invasion assay. *∗∗*:* P*<0.01; *∗∗∗*:* P*<0.001 versus control group.

Since the relentless invasion of malignant gliomas is the major cause resulting in poor outcome and death of glioma patients. Wound healing assay showed that miR-29a inhibited migration of A172 cells and inhibition of miR-29a enhanced migration ability of U251 cells (Figure 3(a)). Transwell invasion assay showed that miR-29a significantly suppressed invasion of A172 cells and inhibition of miR-29a promoted invasion of U251 cells (Figure 3(b)). These results collectively indicated a tumor suppressing role of miR-29a in malignant gliomas.

### 3.3. MiR-29a Directly Targets TRAF4 and Akt Activation

**Figure 4:**
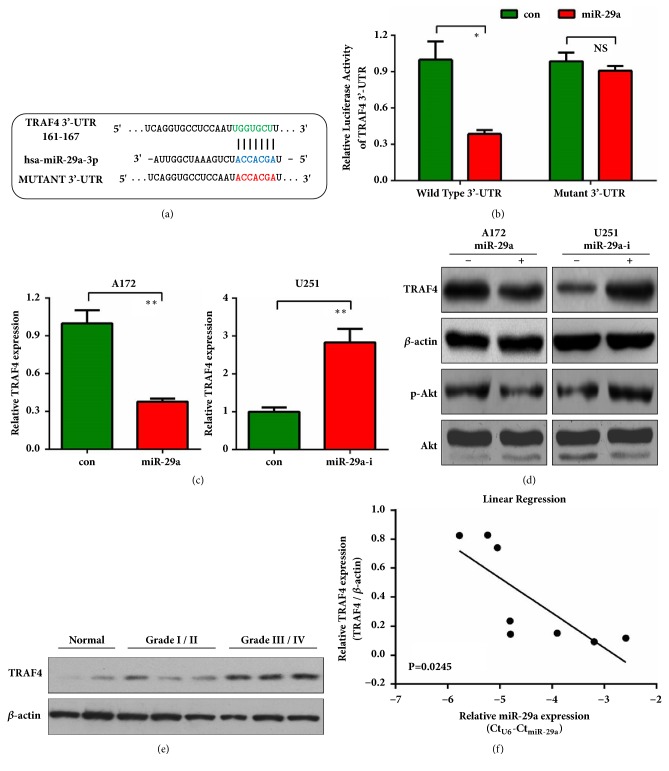
**MiR-29a-3p directly targets TRAF4. (a)** The wild-type 3'-UTR of TRAF4 contains one predicted miR-29a-3p binding site in green. The mutagenesis nucleotides are indicated in red.** (b)** Dual-luciferase reporter assay. A172 cells were transfected with wild-type 3'-UTR-reporter or mutant 3'-UTR reporter together with control or miR-29a mimics. Relative firefly luciferase expression was normalized to renilla luciferase.** (c)** A172 or U251 cells were transfected with miR-29a mimics or inhibitors. 48h later, cells were harvested for qRT-PCR to detect the mRNA level of TRAF4.** (d)** Protein expression analysis of TRAF4 and p-Akt was performed by western blot in A172 and U251 cells treated as** (c)**.** (e)** Protein expression analysis of TRAF4 was performed by western blot in noncancerous brain tissues and glioma samples with different tumor grades (Grade I+II and Grade III+IV).** (f)** Linear correlation between miR-29a and TRAF4 expressions in noncancerous brain tissues and glioma samples. Statistical analysis was performed by Pearson's correlation analysis. *∗*:* P*<0.05; *∗∗*:* P*<0.01 versus control group.

To further understand the mechanism of miR-29a in glioma, we used TargetScan, miRTarBase, and PicTar to predict the putative targets of miR-29a. All the three tools revealed that the 3'-UTR of TNF receptor-associated factor 4 (TRAF4) mRNA contained a conserved miR-29a target region (Figure 4(a)). A series of experiments were then performed to confirm our prediction. Specific targeting of TRAF4 by miR-29a was examined using luciferase reporter assays. As shown in Figure 4(a), a reporter plasmid containing mutant 3'-UTR of TRAF4 was constructed. Introduction of miR-29a significantly repressed wild-type 3'-UTR of TRAF4; however, it failed to repress the mutant reporter (Figure 4(b)), indicating a direct targeting of TRAF4 by miR-29a. Next qRT-PCR and western blot further confirmed that TRAF4 mRNA and protein levels were negatively regulated by miR-29a in A172 and U251 cells (Figures 4(c) and 4(d)). As a transducer of Akt signaling [20], we observed that, similar to TRAF4, phosphorylated Akt (p-Akt) was also inhibited by miR-29a (Figure 4(d)). Then we tried to detect the correlation between expression levels of TRAF4 and miR-29a in the above mentioned noncancerous brain tissues and glioma samples. Western blot results showed that TRAF4 was higher in glioma samples compared to noncancerous tissues, keeping on increasing with the elevation of tumor grade (Figure 4(e)). Moreover, TRAF4 expression in these tissues was negatively correlated with miR-29a expression (Figure 4(f)). These results reveal that miR-29a may directly bind with TRAF4 3'-UTR and inhibit TRAF4 mRNA/protein expression thus repressing Akt signaling in glioma cells.

### 3.4. TRAF4/Akt Induces MiR-29a Expression in GBM Cells

**Figure 5:**
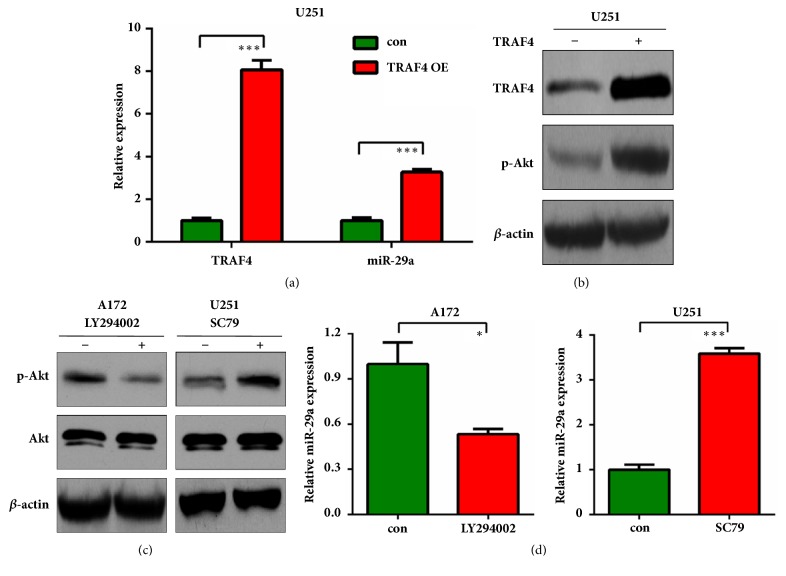
**TRAF4 regulates the expression of miR-29a. (a) **U251 cells were transfected with TRAF4 overexpressing plasmid. 24h later, cells were harvested for qRT-PCR to detect the expression level of TRAF4 and miR-29a.** (b)** Protein expression analysis of TRAF4 and p-Akt was performed by western blot in U251 cells treated as** (a)**.** (c)** A172 or U251 cells were treated with 20*μ*M LY294002 or 5*μ*g/ml SC79 for 16h. Cells were harvested for western blot to detect the protein level of p-Akt.** (d)** mRNA expression analysis of miR-29a was performed by qRT-PCR in A172 or U251 cells treated as** (c)**. *∗*:* P*<0.05; *∗∗∗*:* P*<0.001 versus control group.

To better understand the interplay of TRAF4 and miR-29a in gliomas, we investigated whether TRAF4 or Akt activation might exert some effects on the tumor suppressor miR-29a. TRAF4 expressing plasmid was transfected and transfection efficiency was confirmed (Figure 5(a)). We found that miR-29a level was elevated by TRAF4 overexpression (P<0.001; Figure 5(a)). Meanwhile, TRAF4 overexpression also induced Akt activation as assessed by western blot of p-Akt (Figure 5(b)). We then used Akt inhibitor LY294002 and agonist SC79 to treat A172 and U251 cells, respectively. After validating the inhibition or activation of Akt signaling in the two cell lines (Figure 5(c)), we surprisingly found that treatment by Akt inhibitor inhibited miR-29a level, whereas treatment by Akt agonist enhanced miR-29a level in the tested cells (Figure 5(d)), suggesting that miR-29a could be directly induced by TRAF4/Akt signaling. These results reveal a negative feedback loop between miR-29a and TRAF4/Akt signaling in glioma.

## 4. Discussion

It is known that miR-29a is an important tumor suppressor in several human cancers including in gliomas [16–18]. Specifically, the decreased miR-29a expression is closely correlated with aggressive phenotypes and shorter survival of glioma patients [18, 19]. We learned from others' reports that miR-29a might target different genes and act through them to act in tumorigenesis [17]. Although several targets have been identified for miR-29a in glioma [18, 19, 21], we still should integrate more targets into the true function of miR-29a in glioma. In the present study, we identified miR-29a as a downregulator and a tumor suppressor to inhibit the cell proliferation, migration and invasion in gliomas. Mechanistically, we verified TRAF4 as a direct target of miR-29a in gliomas, which could further mediate Akt pathway activation. This finding improved our understanding of the mechanisms underlying miR-29a-repressed glioma progression. Our results collectively highlighted the potential values of miR-29a as a novel therapeutic target in human gliomas.

Tumor necrosis factor receptor-associated factors (TRAFs) are initially discovered as adaptor proteins that regulate cell life and death [22]. So far, seven members (TRAF1-7) have been identified, and TRAF4 is a special member of the TRAF family: it contains a nuclear localization signal (NLS) and three CART domains (Cystein-Rich domain Associated with RING and TRAF domain) [23]. TRAF4 was firstly identified in breast carcinomas and represents the first member of the TRAF family to be increased and expressed in human cancers [23]. As an adaptor, TRAF4 is likely to be involved in signal transduction since it directly or indirectly mediates the downstream signal molecules. The serine/threonine kinase Akt is a central node in cell signaling downstream of growth factors, cytokines, and other cellular stimuli [24]. As mentioned in other cancers that TRAF4 upregulation promoted the activation of the Akt signaling pathway [25, 26], we also observed that TRAF4 expression positively correlated with p-Akt level. We herein observed that TRAF4 or Akt stimulation induced miR-29a expression and Akt blockage repressed miR-29a expression. We considered that miR-29a repressed TRAF4/Akt activation, but conversely, TRAF4/Akt induced miR-29a to further targeting TRAF4/Akt pathway. This forms a negative feedback to control TRAF4/Akt levels in glioma.

In summary, our study identified that miR-29a plays an inhibitory role in glioma by suppressing cell proliferation, migration, and invasion. We also validated TRAF4 as a direct target of miR-29a. Our study contributes to clarify the tumor suppressing function of miR-29 through forming a negative feedback loop of TRAF4/Akt signaling, and miR-29a represents a potent therapeutic target for treating gliomas.

## Data Availability

The data used to support the findings of this study are available from the corresponding author upon request.
